# Evaluation of Anti-Inflammatory Activities of Qingre-Qushi Recipe (QRQS) against Atopic Dermatitis: Potential Mechanism of Inhibition of IL-33/ST2 Signal Transduction

**DOI:** 10.1155/2017/2489842

**Published:** 2017-07-02

**Authors:** Mengjiao Chen, Peijun Ding, Lili Yang, Xufeng He, Chunjie Gao, Guoxun Yang, Huimin Zhang

**Affiliations:** ^1^Department of Dermatology, Shuguang Hospital Affiliated to Shanghai University of Traditional Chinese Medicine, Shanghai 200000, China; ^2^School of Pharmacy, Fudan University, Shanghai 200000, China

## Abstract

To evaluate the anti-inflammatory activities of QRQS against AD and the inhibitory molecular mechanisms of IL-33/ST2 signal transduction, BALB/c mice were divided into six groups (normal control, OVA control, low-dose of QRQS, middle-dose of QRQS, high-dose of QRQS, and cetirizine) and epicutaneously exposed to ovalbumin or PBS for 3 weeks and treated with QRQS for 2 weeks. Skin biopsies and blood samples were obtained for histological study, antibody analysis, and RNA isolation. HaCaT cells, stimulated by TNF-*α* and IFN-*γ*, were treated with QRQS to evaluate mRNA and protein expression by RT-PCR and ELISA. QRQS decreased both epidermal and dermal thickness, alleviated dermatitis, and reduced IL-33 and ST2 positive cell numbers. The concentration of specific IgE, IgG, IgG1, and IgG2a antibodies in serum and the expression of IL-33, ST2, IL-1RAcP, IL-4, and IL-13 mRNA in the skin were suppressed. No significant difference exists in TNF-*α* or IFN-*γ*. QRQS decreased IL-33 mRNA and protein secretion in HaCaT cells exposed to TNF-*α* and IFN-*γ* in a time- and concentration-dependent manner. QRQS regulates related molecule expression of ovalbumin-induced dermatitis involved in the IL-33/ST2 signaling axis in the treatment of acute AD.

## 1. Introduction

Atopic dermatitis (AD) that mainly begins in early life is one of the most common chronic inflammatory skin disorders characterized by xerosis cutis, inflamed skin, excoriation, and crusting. It is marked by an increased ability to form reagin (IgE), especially the allergen-specific IgE, in the sera, with increased susceptibility to allergic rhinitis and asthma, as well as a hereditary disposition to a lower threshold for pruritus [1, 2]. AD mainly affects children [3], and, in infants, it is known as infantile eczema. Nearly 60% of the cases consist of AD outbreak within the first year of life and up to 95% begin before the age of five [4, 5].

Previous studies have shown that interleukin-33 (IL-33) is highly expressed in skin keratinocytes and endothelial cells, and its effect on the skin may be related to the early stage of skin inflammation [6] and AD. Therefore, the mechanism of the IL-33/ST2 signaling pathway is a potential target for the treatment of AD. IL-33 interaction with the ST2 receptor leads to the activation and recruitment of MyD88 adapter protein along with IL-1R-associated kinase1 (IRAK1), IRAK4, and TNFR-associated factor 6 (TRAF6). This signaling cascade further leads to the activation of transcription factors such as NF-*κ*B and MAP kinases and the production of inflammatory mediators. MyD88 is crucial for several functional responses to IL-33, such as survival cytokines' production and mast cells (MCs) proliferation. IL-33 treatment further leads to the activation of different kinases such as ERK1/2, p38MAPK, and JNK. IL-33 mediated signaling pathways further modulate MC functions. IL-33 has also been reported to activate MCs and act directly on Th2 cells to increase the secretion of Th2 cytokines such as IL-4 and IL-13. Furthermore, IL-33 functions as a chemoattractant for Th2 cells [7].

QRQS oral liquid is a herbal formulation containing four herbs according to the Traditional Chinese Medicine (TCM) theory, including* Hedyotis diffusa* Willd.,* Xanthium sibiricum*,* Taraxacum*, and* Sophora flavescens* Ait. Based on TCM theory, these herbs are heat-clearing and dampness-dispelling. QRQS has been used as a medicinal formula to alleviate skin inflammation and itching in traditional herbal medicine [8–10]. The biological activities of each herb have been reported as follows: Taraxacum has been confirmed to have pharmacological activities of anti-inflammatory, antimicrobial (antibacterial, antifungal), and antiviral activity [11].* Hedyotis diffusa* Willd. has been reported to have anti-inflammatory activity via suppression of the NF-*κ*B and MAPK signaling pathways [12]. Ju et al. [13] demonstrated that* Xanthium sibiricum* has an anti-inflammatory property in LPS-stimulated RAW 264.7 macrophages, and the activity is exerted by the regulation of NF-*κ*B and STAT3 signaling pathways. Recent studies indicated that a minimum of 50 pure compounds and crude extracts from* Sophora flavescens* possess wide-ranging antitumor, antimicrobial, and anti-inflammatory pharmacological abilities [14]. Other studies have reported that IL-33 is involved in the inflammatory response of AD [6, 15]. However, the therapeutic potential of QRQS for AD through Th2-mediated IL-33/ST2 inflammatory response has not been investigated. In the present study, we explored whether oral administration of QRQS inhibits AD in ovalbumin-induced AD model of BALB/c mice by IL-33/ST2 signal transduction.

## 2. Materials and Methods

### 2.1. Preparation of QRQS

*Hedyotis diffusa* Willd.,* Taraxacum*, Xanthium sibiricum, and* Sophora flavescens* Ait. contained in QRQS were purchased from Shuguang Hospital Affiliated with the Shanghai Chinese Medicine University. To maintain consistency among the herbal ingredients, all of the herbal components were initially obtained from standard sources as stated within the Chinese GAP grade. QRQS was prepared as follows: The four herbs (4 : 4 : 1 : 4) were soaked in water (1 : 10 w/v) and boiled at 100°C twice, 1 h each time. The boiled herbs were then filtered through a Whatman number 2 filter paper (Maidstone, UK), concentrated under vacuum conditions, and freeze dried. The extract was stored at −80°C, determined by a hydrometer (Beijing Heng Odd Instrument Co., Ltd., China) and dissolved in phosphate-buffered saline (PBS) before use.

### 2.2. HPLC Conditions

**Figure 1:**
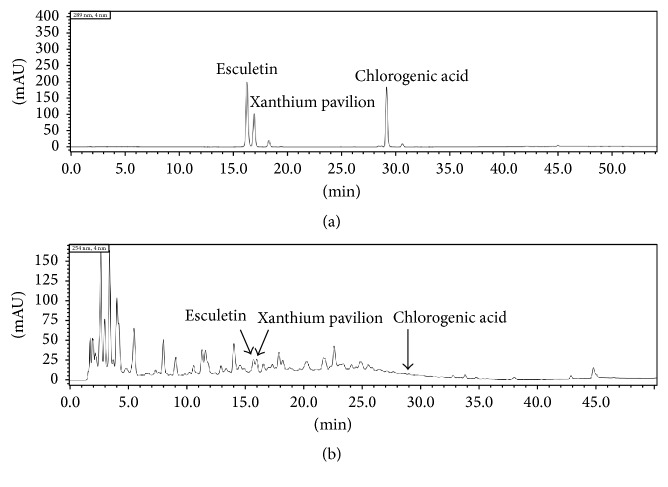
HPLC chromatogram of three index components and QRQS sample. (a) Representative HPLC chromatogram of three index components solution. (b) The chromatogram of three index components in QRQS sample.

The HPLC system consisted of a SHIMADZU LC-20AT chromatography and a SPD-M20A detector. 0.1 mg Esculetin, 0.1 mg Xanthium Pavilion, and 1.2 mg chlorogenic acid were dissolved in 2 ml methanol and filtered in millipore filter. The QRQS sample was analyzed after 10 times dilution. Chromatographic separation was conducted on a Dikma C18 (4.6 mm × 150 mm, 5 *μ*m). The mobile phase was methanol/water in a gradient elution, produced by starting at 10 : 90 v/v, changing to 90 : 10 v/v over 35 min of elution, then gradually to 100 : 0 v/v reached at 40 min. This was maintained until 50 min. The flow rate was 1 mL/min, and the injection volume was 20 *μ*L (Figure 1).

### 2.3. Animal Experiment

Specific pathogen-free 6-week-old female BALB/c mice were purchased from Chinese Academy of Sciences (Shanghai, China) and maintained under pathogen-free conditions. Mice were acclimated for 1 week to the housing condition before the start of the experiments. Subsequently, 5 mice/cage were housed in a laminar air flow room with a relative humidity of 55 ± 5%. Each room was maintained at 22 ± 2°C on a light-dark cycle of 12 h light and 12 h dark throughout the experiment. The sensitization protocol was performed as described previously [16]. Briefly, after anesthesia with isoflurane inhalation (Ren Yi Biological Science and Technology Co., Ltd., Shanghai, China), the back hair of the mice was shaved and then stripped 6 times by a hyaline adhesive tape (Tegaderm, 3M Health Care, St. Paul, MN, USA) to introduce a standardized skin injury. A sterile gauze patch (1 × 1 cm^2^) containing 100 *μ*L of 0.1% ova-albumin (OVA group) or 100 *μ*L PBS (PBS group) was placed on the back skin and secured with Tegaderm. The patches were maintained for 1 week and the whole experiment comprised a total of three 1-week exposures with 2-week intervals between each exposure week.

**Figure 2:**
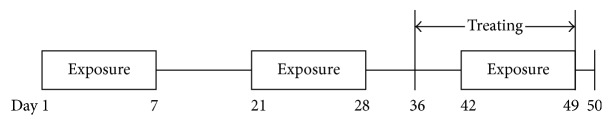
Sensitization protocol. BALB/c mice were exposed to OVA (100 *μ*g/mL) or PBS applied in 100 *μ*L to a sterile patch. The experiment comprised a total of three 1-week exposures separated by a 2-week resting interval. Medicines were administered from the 36th day lasting for 2 weeks. All tests were performed at the end of the third sensitization, and the blood and skin biopsies were taken.

Treated mice were divided into six groups (10–12 per group; NC: normal control; MC: model control; LD: low-dose QRQS; MD: middle-dose QRQS; HD: high-dose QRQS; Ceti: cetirizine) and were orally treated with PBS, QRQS (4, 2, or 1 g/kg/day), or cetirizine (1.3 mg/kg/day, Bright Future Pharmaceuticals Factory) every day for 2 weeks. The naïve group was treated with vehicle (PBS) on the dorsal skin and orally given PBS. The severity of dermatitis was assessed once a week by three persons blinded to the identities of the groups, according to the method described by Leung et al. [17]. A total clinical index of dermatitis severity was defined as the sum of the individual scores graded as follows: 0 (none), 1 (mild), 2 (moderate), and 3 (severe) for each of the five signs and symptoms (erythema/hemorrhage, edema/hematoma, excoriation/erosion, itching/dryness, and lichenification [thickness of the skin]). The scratching frequency was measured by counting the number of times that mice scratched the body using the hind paws during a 20 min period. A blood sample was withdrawn for antibody analysis. Skin biopsies from treated areas were obtained for RNA isolation, histology, and immunohistochemistry (Figure 2).

### 2.4. Ethics Statement

Animal care and manipulation were in agreement with institutional guidelines, which are in accordance with the Guide for the Care and Use of Laboratory Animals. All animal experiments were approved by the Animal Experiment Center of Shanghai University of TCM. All the surgeries were performed under isoflurane inhalation (Shanghai Ren Yi Biological Science and Technology Co., Ltd.), and all efforts were made to minimize the suffering of the animals (Table 1).

### 2.5. Cell Culture

HaCaT cells were purchased from Shanghai Bo Valley Biological Technology Co., Ltd. and cultured in Dulbecco's Modified Eagle Medium (DMEM, Life Technologies Corporation), supplemented with 10% heat-inactivated fetal bovine serum (FBS), penicillin (100 U/mL), and streptomycin (100 *μ*g/mL) (Gibco Inc.), in a 5% CO_2_ incubator at 37°C. At approximately 80–90% confluency, the cells were trypsinized, diluted in a ratio of 1 : 4, and passaged. Cells passaged 3–6 times were used for this study. HaCaT cells were stimulated with 50 ng/mL of TNF-*α* or a combination of 50 ng/mL TNF-*α* and 50 ng/mL IFN-*γ* for 24 h for RNA isolation. Subsequently, after 24, 32, 48, and 56 h, the liquid supernatant was collected for ELISA.

### 2.6. ELISA

Total IgG, IgG1, IgG2a, and OVA-specific IgE were estimated by the direct ELISA method as described previously [16]. Briefly, the plates were coated with 100 *μ*g/mL OVA in 0.05 M NaHCO3 (pH 9.6) at 4°C overnight. The plates were then washed with PBS-Tween 20 (0.05%) and blocked with PBS-3% bovine serum albumin (BSA) for 2 h at 20°C and washed again. 100 *µ*L of diluted sera (1 : 500 for IgG and IgG1, 1 : 100 for IgG2a, 1 : 50 for IgE) in 1% BSA-PBS was incubated at 4°C overnight. After washing, 2 *μ*g of biotin-conjugated rat anti-mouse IgE mAb (clone R35-118) in 1 mL 1% BSA-PBS was incubated at 4°C overnight. Subsequently, after washing, 2 *μ*g of substrate was incubated for 2 h at 20°C and washed again. Streptavidin-horseradish peroxidase (BD PharMingen) and peroxidase substrate reagents (Kirkegaard & Perry Laboratories, Gaithersburg, MD, USA) were used to develop the colored reaction. The absorbance was measured at 405 nm with an automated ELISA reader (PowerWave XS2, BioTek, USA).

### 2.7. RNA Extraction, Reverse Transcription, and RT-PCR

Total cellular RNA was isolated using TRIzol (Ambion, life technologies) and quantified at 260 nm. The complementary DNA was synthesized from 1–5 *μ*g RNA using a HiFi-Script cDNA First Synthesis Kit (CWBIO) according to the manufacturer's protocol. Primers for GAPDH, IFN-*γ*, IL-1RAcP, TNF-*α*, ST2, IL-14, IL-13, and mouse and human IL-33 were obtained from Invitrogen. RT-PCR was performed on a Real-Time PCR System (Applied Biosystems, USA) using the SYBR Green RT-PCR Kit (Thermo Scientific) according to the manufacturer's instructions.

Quantification was performed with a two-step reaction process: reverse transcription (RT) and PCR. Each RT reaction consisted of 500 ng RNA, 2 *μ*L PrimerScript Buffer, 0.5 *μ*L oligo dT, 0.5 *μ*L random mers, and 0.5 *μ*L PrimerScript RT Enzyme Mix I (TaKaRa Bio, Japan), in a total volume of 10 *μ*L. Reactions were performed in a PCR system for 15 min at 37°C, followed by heat inactivation for 5 s at 85°C. The reaction was then diluted 10-fold in nuclease-free water and stored at −20°C. The 10 *μ*L PCR reaction mixture included 1 *µ*L cDNA, 5 *μ*L 2x SYBR Green I Master (Roche, Swiss), 0.2 *μ*L forward primer, 0.2 *μ*L reverse primer, and 3.6 *μ*L nuclease-free water. Reactions were incubated in an optical plate (Roche, Swiss) at 95°C for 10 min, followed by 40 cycles of 95°C for 10 s and 60°C for 30 s. At the end of the PCR cycle, melting curve analysis was performed to validate the specific PCR product. The primer sequences were designed in the laboratory and synthesized by Generay Biotech (Generay, China), based on the mRNA sequences obtained from the NCBI database. Primers used for PCR amplification are listed in Table 1. The expression levels of mRNAs were normalized to GAPDH and were calculated using the 2^−ΔΔCt^ method.

### 2.8. Histology and Immunohistochemistry (IHC)

The mice were anesthetized by isoflurane inhalation and executed before harvesting the skin samples. The skin samples from the lesion area were fixed in 10% formaldehyde for 24 h and embedded in paraffin for sectioning. The skin sections were stained with hematoxylin and eosin (HE). The thickness of the epidermal, dermal, and various inflammatory cells was analyzed from HE-stained sections and visualized under a magnification of ×200. Five fields from each sample were randomly selected to measure the thickness of epidermis and dermis.

Immunoperoxidase staining was used to detect IL-33 and ST2-positive cells in the sensitized skin. Sections of 4 mm were prepared and stained by rabbit HRP-DAB (ABC detection IHC kit, Abcam). Briefly, endogenous peroxidase activity was blocked by peroxidase blocking solution and protein block was applied to exclude the nonspecific staining. Then, the sections were incubated with biotinylated goat polyclonal antibody, followed by streptavidin-peroxidase at room temperature. The sections were then stained in DAB substrate for 1 min. The tissue sections were mounted with Aquamount (BDH, Gurr, Pole, UK). The positively stained IL-33 (1.25 *μ*g/mL; ab118503; Abcam) and ST2 (0.75 *μ*g/mL; ab25877; Abcam) cells were counted in 10–15 high power fields (HPFs) at ×400 and expressed as cells/HPF, with mean and SE.

## 3. Statistical Analysis

The data are expressed as the means and standard error (SE). SPSS 18.0 software (SPSS Inc., Chicago, USA) was used for statistical analysis. The significance of the differences was determined by one-way ANOVA; comparisons of parameters between two groups were performed with LSD or Games-Howell test. The RT-PCR data for gene expression levels in normal mice was set at 1-fold; the altered gene expressions of the other groups were expressed as the normalized “fold” change compared with the normal mice. Appropriate analyses of variance were also performed to confirm significant differences between the groups. *P* < 0.05 was considered statistically significant.

## 4. Results

### 4.1. Effects of QRQS on the Treatment of Alleviating AD-Like Symptoms

**Figure 3:**
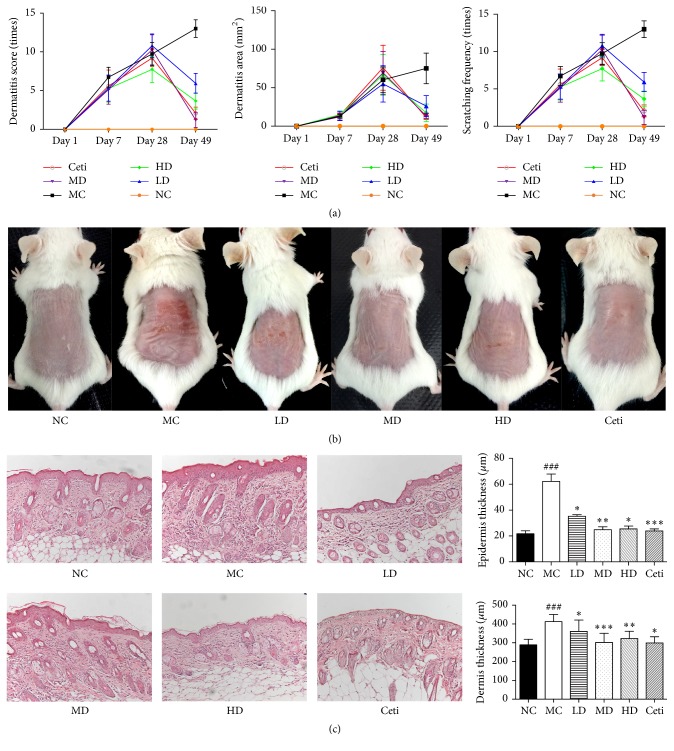
Effects of QRQS on the development of AD in BALB/c mice. (a) Dermatitis score, dermatitis area, and scratching frequency are significantly decreased after treatment with QRQS as compared to the model control. (b) Dermatitis on BALB/c mice dorsal skin. (c) H&E sections from each group. Represented sections are shown at ×200 magnification. Data are expressed as means ± SEMs for each group of 10 rats. Asterisks denote a significant difference: ^*∗*^*P* < 0.05, ^*∗∗*^*P* < 0.01, and ^*∗∗∗*^*P* < 0.001 for QRQS-treated group versus MC group; ^###^*P* < 0.001 for MC group versus normal group.

To determine whether QRQS can relieve local inflammation in the skin sensitized by OVA, the alterations in the dermatitis score, dermatitis area, and scratching frequency were recorded on days 1, 7, 28, and 49, respectively. There was a significant increase in all the three parameters in the OVA-treated group. However, a significant decrease was observed in each after the treatment of low-dose, middle-dose, high-dose QRQS, and cetirizine. Biopsies from each group of mice were taken 1 d after the completion of the third series of sensitization. We found thickening and inflammation in the dermis and epidermis at the site of OVA sensitization but not with each treatment group. The epidermal layer was approximately 3-fold thick in OVA-sensitized sites compared with the normal control and the middle-dose QRQS (Figure 3).

### 4.2. Effects of QRQS on OVA-Specific IgE, IgG, IgG1, and IgG2a

**Figure 4:**
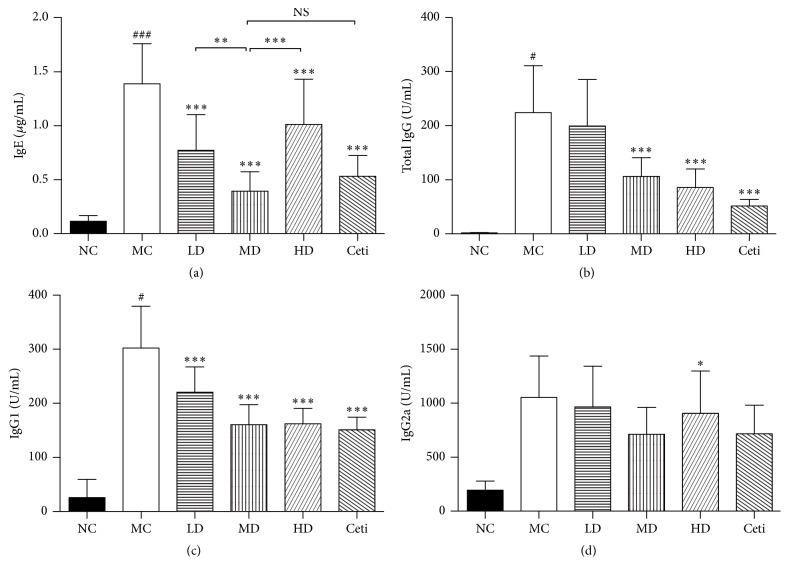
Changes in the antigen-specific antibody response in mice after treatment with QRQS or cetirizine (*n* = 10 for each). All the above antigens were increased after OVA induction and decreased after treatment with QRQS and cetirizine. The level of antibodies was determined by ELISA. The data are shown as mean ± SEMs. NS: not statistically significant; ^#^*P* < 0.05, ^###^*P* < 0.001, MC compared with NC; ^*∗*^*P* < 0.05, ^*∗∗*^*P* < 0.01, and ^*∗∗∗*^*P* < 0.001, as compared to MC by LSD test.

Different concentrations of QRQS can bring about various degrees of inhibition in the OVA-specific IgE levels in the peripheral blood of AD mice such that QRQS clearly inhibits the therapeutic effect of allergic reactions. Furthermore, QRQS can reduce ova-specific IgG levels in a concentration-dependent manner. In this case, the Chinese medicine dose group, high-dose group, and the Western cetirizine group are significantly different (*P* < 0.001). This indicates that QRQS exerts a definite inhibition of the allergic reactions in the humoral immunity as a result of the disease effect. In addition, QRQS for immunoglobulins of the IgG1 subtypes has different degrees of inhibition, in which the Chinese medicine dose group and the Western cetirizine group of IgG1 inhibition have significant differences (*P* < 0.001). Also, the Chinese medicine group and Western cetirizine group inhibits the IgG1 at a high rate. Model group and the treatment dose group of IgG2a were not statistically significant (Figure 4).

### 4.3. Effects of QRQS on Th1- and Th2-Associated Cytokines Expression

**Figure 5:**
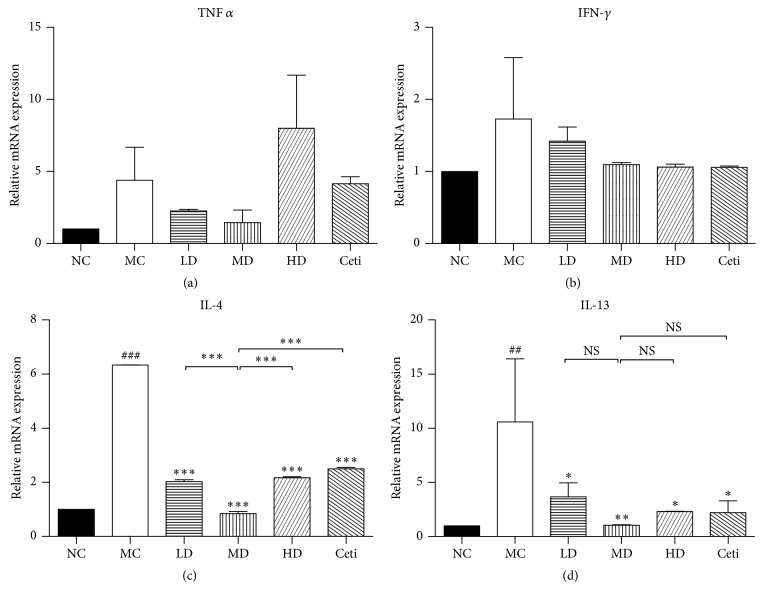
mRNA expression of cytokines in mice skin after treatment with QRQS or cetirizine. RT-PCR was used to analyze the mRNA expression levels. Relative unit (RU) is expressed as fold differences relative to the calibrator. Statistical results were expressed as mean ± SEMs. ^###^*P* < 0.001, ^##^*P* < 0.05, MC compared with NC; ^*∗*^*P* < 0.05, ^*∗∗*^*P* < 0.01, and ^*∗∗∗*^*P* < 0.001 compared with MC; NS: not statistically significant.

In the present study, we found that after OVA sensitization, IFN-*γ* mRNA levels in the back skin of the model control group mice were rising, but without statistical significance. Also, no statistical significance was observed between different concentrations of QRQS and the positive control group (*P* > 0.05). Compared to the normal control, TNF-*α* mRNA levels in the model group have increased, while there was no significant difference among low-dose QRQS group, middle-dose QRQS group, high-dose QRQS, and cetirizine group (*P* > 0.05). IL-4 mRNA levels of the model control group increased significantly compared with the normal control group (*P* < 0.001). IL-4 levels of mRNA were significantly decreased among the different concentrations of QRQS decoction and cetirizine group (*P* < 0.001). IL-13 mRNA level was increased substantially in the model control group compared with the normal control group (*P* < 0.01) and decreased with different concentrations of QRQS and cetirizine treatment groups. Among them, the mRNA IL-13 level of QRQS was lower than that of the cetirizine group (Figure 5).

### 4.4. Effects of QRQS on the Expression of IL-33 and Its Receptors IL-1RAcP and ST2

**Figure 6:**
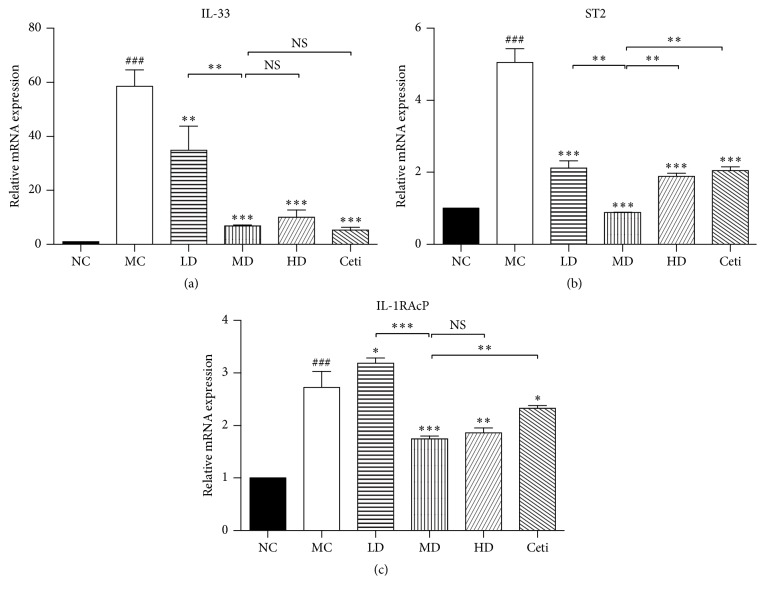
The effects of different concentrations of QRQS decoction on the expression of IL-33 mRNA (a) and its receptor IL-1RAcP (b), ST2 (c). Statistical results were expressed as mean ± SEMs, ^###^*P* < 0.001, compared with the normal group; ^*∗*^*P* < 0.05, ^*∗∗*^*P* < 0.01, and ^*∗∗∗*^*P* < 0.001 compared with the model group.

**Figure 7:**
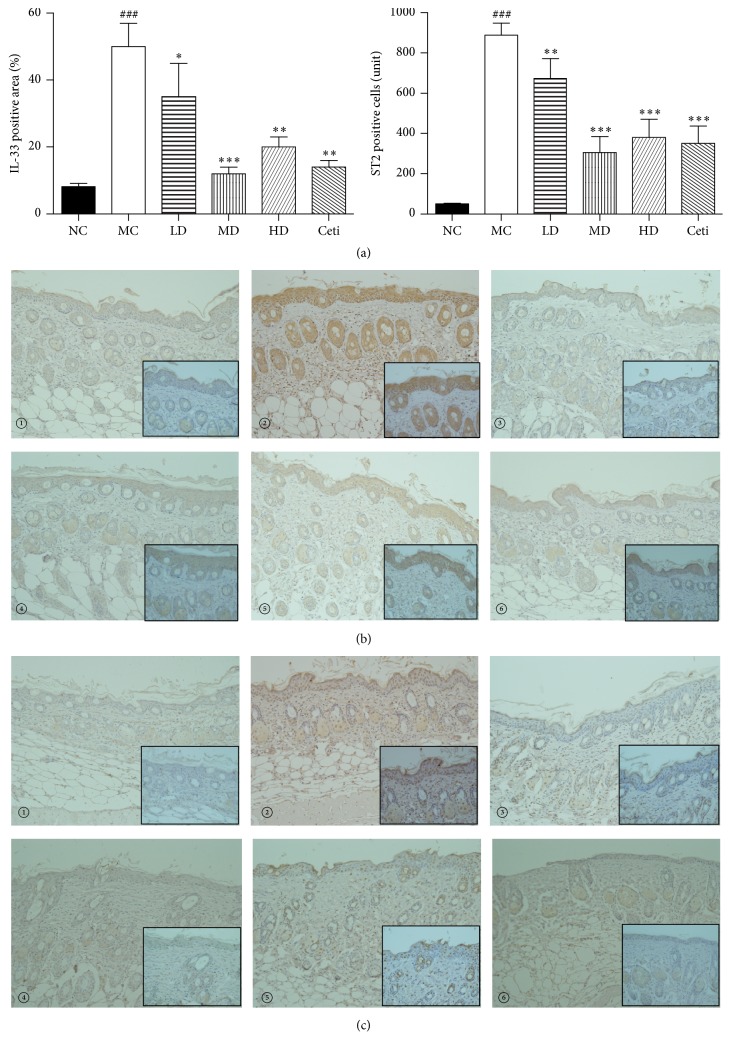
(a) Results of immunohistochemical detection of IL-33 positive area and ST2 positive cells (×200). High magnification photo is in the black square frame (×400). Statistical results were expressed as mean ± SEMs, ^###^*P* < 0.001 compared with the normal group; ^*∗*^*P* < 0.05, ^*∗∗*^*P* < 0.01, and ^*∗∗∗*^*P* < 0.001 compared with the model group. (b) Detection of the expression of IL-33 protein in mouse dorsal skin tissue by immunohistochemistry. (c) Detection of the expression of ST2 protein in mouse dorsal skin tissue by immunohistochemistry. (①): normal control group; (②): model control group; (③): low-dose QRQS group; (④): middle-dose QRQS group; (⑤): high-dose QRQS group; (⑥): cetirizine medicine group.

The RT-PCR assessment of the lesion on the skin of the experimental animal model of AD demonstrated that the secretion of IL-33 in skin tissue of the model group was significantly higher than that in the normal group. Moreover, different concentration treatment groups show different degrees of improvement, especially in the middle-dose group; the improvement degree of the treatment group is similar to that of the cetirizine group. IL-33 and its receptors ST2 and IL-1RAcP model mice lesions also increased significantly. Interestingly, the different doses of QRQS decoction in the treatment group on the mouse skin tissue of ST2 receptor have different degrees of inhibitory effect; the dose group compared with the positive drug cetirizine in the treatment group improved significantly. IHC showed that the model group showed thickening of the epidermis, and IL-33 and ST2 positive cells showed a marked expression. In the Chinese medicine group of IL-33 and ST2 positive cells, the expression is lower than that in the model group, especially in the middle-dose Chinese medicine group and cetirizine group, suggesting that QRQS plays an excellent role in the regulation of IL-33 and ST2 protein expression (Figures 6 and 7).

### 4.5. Effects of QRQS Treatment on IL-33 in HaCaT Cells

**Figure 8:**
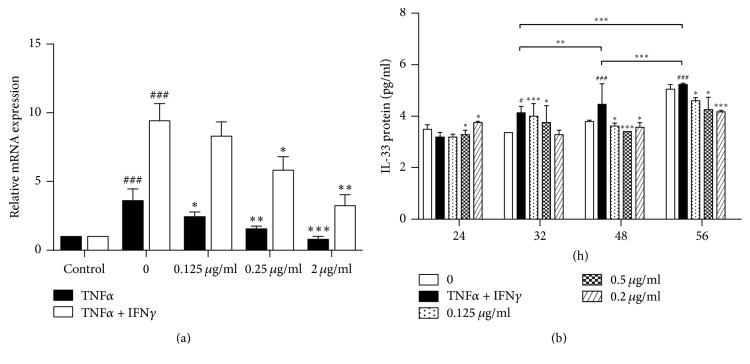
(a) Effect of QRQS on TNF-*α*– or TNF-*α* +IFN-*γ*-induced HaCaT cells to produce IL-33 mRNA and protein. TNF-*α* (50 ng/mL) or TNF-*α* (50 ng/mL) + IFN-*γ* (50 ng/mL) stimulated HaCaT cells were cultured with different concentrations of QRQS solution for 24 h. RT-PCR was used to detect the IL-33 mRNA secretion. (b) The influence of different time points in different concentration decoctions on the expression of the IL-33 protein. The TNF-*α* (50 ng/mL) + IFN-*γ* (50 ng/mL) combined with HaCaT cells were treated with different concentrations (0.125 g/mL, 0.5 g/mL, and 2.0 g/mL) of QRQS, and, at different time points (24, 32, 48, and 56 h), supernatants were collected. The IL-33 protein expression was detected by ELISA. The data are shown as mean ± SEMs, ^###^*P* < 0.001, compared with the blank group; ^*∗*^*P* < 0.05, ^*∗∗*^*P* < 0.01, and ^*∗∗∗*^*P* < 0.001, compared with the model group.

Previous studies found that the expression of TNF-*α* and IFN-*γ* can induce the expression of IL-33 to promote allergic dermatitis [18]. Herein, we used in vitro experiments to investigate whether QRQS can inhibit TNF-*α*– and IFN-*γ*-induced IL-33. RT-PCR results show that (1) compared with and without the addition of stimulators of normal group, the addition of 50 ng/mL TNF-*α* significantly stimulated the HaCaT cells producing IL-33 mRNA (*P* < 0.001). (2) Compared with the model group, the addition of the different concentration of QRQS can significantly reduce the HaCaT cells producing IL-33 mRNA levels (*P* < 0.05, *P* < 0.01, or *P* < 0.001). (3) Compared with the normal group, the addition of 50 ng/mL TNF-*α* + 50 ng/mL IFN-*γ* significantly stimulated HaCaT cells producing IL-33 mRNA (*P* < 0.001). (4) Compared with the model group, adding a different concentration of QRQS significantly reduced the HaCaT cells producing IL-33 mRNA levels (*P* < 0.05 or *P* < 0.01), whereas low levels of QRQS group decreased the IL-33 mRNA levels, but not remarkably. These results show that the QRQS recipe (0.125 g/mL, 0.25 g/mL, 2 g/mL) inhibited the HaCaT cells induced by TNF-*α* or TNF-*α* and IFN-*γ* of IL-33 mRNA in a concentration-dependent manner. RT-PCR results demonstrated that, under a combined stimulation of TNF-*α* (50 ng/mL) + IFN-*γ* (50 ng/mL), HaCaT cells can secrete higher IL-33 mRNA; thus, we used TNF-*α* + IFN-*γ* combined stimulation. The results showed that TNF-*α* (50 ng/mL) + IFN-*γ* (50 ng/mL) combined stimulation for 24 h, 32 h, 48 h, and 56 h gradually increased the protein expression of IL-33 in a time-dependent manner (*P* < 0.01 or *P* < 0.001). When QRQS and HaCaT cells were cocultured for 24 h, the model group did not display an increased IL-33 protein expression, and the different doses of QRQS group showed no significant variations (*P* > 0.05). 32 h post-TNF-*α* and IFN-*γ* stimulation, the stimulated model shows that IL-33 protein expression was significantly increased (*P* < 0.05) and the QRQS groups was dose-dependent. Moreover, the low-dose group (0.125 g/mL) and the middle-dose group (0.5 g/mL) differed considerably (*P* < 0.001 or *P* < 0.05), whereas the high-dose group showed no statistical significance. After 48 and 56 h, the TNF-*α*– and IFN-*γ*-stimulated model demonstrates that IL-33 protein expression was significantly increased (*P* < 0.001) and various TCM groups were dose-dependent (*P* < 0.05 or *P* < 0.001, Figure 8).

## 5. Discussion

AD is often accompanied by allergic inflammation, which is initiated by the activation of the adaptive immune response. Recent studies have shown that IL-33/ST2-induced immune regulation may have a crucial role in adaptive as well as innate immune responses in skin [19]. Corticosteroids and immunosuppressive drugs, such as tacrolimus, are widely administered to treat AD disease from the local medication but have side effects [20], and novel therapeutics targeting the mediators have not yet been established. QRQS has been used as a medicinal formula to treat allergic reaction-related diseases such as acute dermatitis and eczema in China for a long time [8–10]. Recently studies have shown that oxymatrine and taraxasterol which are the main components of* Sophora flavescens* Ait. and* Taraxacum,* respectively, in QRQS may effectively ameliorate the progression of asthma, the allergic reaction-related disease. It was reported that oxymatrine and taraxasterol could reduce the production of IL-4, IL-5, and IL-13 in bronchoalveolar lavage fluid and OVA-specific IgE in sera and inhibit OVA-induced eosinophilia in lung tissues [21, 22]. However, the mechanism of QRQS in treating AD remains unknown.

In the present study, we have successfully constructed the AD mouse model as described previously [16] and demonstrated that the oral administration of QRQS did not only alleviate the symptoms of AD (Figure 2) but also induce IgE and IgG responses (Figure 3). Considering the serum dilution factors and ODs in ELISA for the OVA-specific IgG subclasses, the middle-dose oral administration of QRQS reduced the most significant production of IgG1 among the IgG subclasses measured, as it requires a maximum contribution from class-switching and IgE Th2 cytokines (Figure 3). Any significance was not observed in IgG2a, and this could be due to the efficacy of QRQS mainly in the acute phase. Classical Th2 cytokines such as IL-4 and IL-13 are expressed in the acute eczematous lesions, whereas in chronic lesions IFN-*γ*-producing Th1 cells are dominant [6]. We demonstrated that QRQS inhibited the expression of IL-4 and IL-13, but there was no significant difference in IFN-*γ* and TNF-*α*, which suggested that QRQS may mainly be effective in the Th2 immune response. The downregulation of Th2 inflammatory response by QRQS may lead to reduced levels of IgE and other antibodies, as well as, remission of AD symptoms (Figure 4). IL-33, a Th2 cytokine, is a recently described tissue-derived cytokine, which is mainly expressed by cells of barrier tissues, and is known to activate the Th2 lymphocytes [23, 24]. IL-33 signals through a receptor complex consisting of an IL-33-specific receptor, ST2, and a coreceptor, IL-1RAcP [23, 25]. The present study demonstrated that QRQS significantly decreased the IL-33 cytokine and protein in a dose- and time-dependent manner [26], which may lead to the downregulation of Th2 cytokines, IL-4, and IL-13 (Figure 5). Further studies of Th1 and Th2 cell intracellular staining for the detection of IL-4 and IFN-*γ* need to be complemented in order to rule out the interference of other cells secreting IFN-*γ* and IL-4.

TNF-*α* is the most frequently used ligand to induce IL-33 production by epithelial cells. IFN-*γ* in a dose- and time-dependent manner induced IL-33 expression in both protein and mRNA, which contribute to the inflammatory activity of AD [18]. We found that QRQS could significantly downregulate IL-33 mRNA and protein (Figures 6 and 7). However, the related downstream signaling pathway necessitates further investigation.

Several herbs such as* Hedyotis diffusa* and* Taraxacum mongolicum* found in QRQS reportedly have anti-inflammatory activities [27]. However, there is a lack of studies describing that the constituting herbs for QRQS have suppressive effects on IL-33/ST2 signal transduction. Therefore, our future studies will focus on examining the effects of each herb on IL-33/ST2 signal transduction and identifying the active compounds. Since there are limited drugs with low side effects and efficient inhibition of itching, well-controlled clinical studies are warranted to demonstrate the benefits of QRQS in human AD.

## 6. Conclusions

In conclusion, the present study demonstrates that QRQS regulates the related molecular expression of ovalbumin-induced dermatitis involved in the IL-33/ST2 signaling axis in the treatment of acute AD.

## Figures and Tables

**Table 1:** Sequences of the oligonucleotides used in RT-PCR reactions.

Cytokine	Forward primer (5′- 3′)	Reverse primer (5′- 3′)
IL-33	ATTTCCCCGGCAAAGTTCAG	AACGGAGTCTCATGCAGTAGA
ST2	AGAAGCCCCAACTTGAATAAGAC	TCTGATCCACGTACTGTCGAG
TNF-*α*	CTGAACTTCGGGGTGATCGG	GGCTTGTCACTCGAATTTTGAGA
IFN-*γ*	ACAGCAAGGCGAAAAGGATG	TGGTGGACCACTCGGATGA
IL-4	GGTCTCAACCCCCAGCTAGT	GCCGATGATCTCTCTCAAGTGAT
IL-13	CCTGGCTCTTGCTTGCCTT	GGTCTTGTGTGATGTTGCTCA
GAPDH	AGGTCGGTGTGAACGGATTTG	GGGGTCGTTGATGGCAACA
